# Single-cell and spatial transcriptome sequencing uncover a platinum-resistant cluster overexpressed TACSTD2 in high-grade serous ovarian cancer

**DOI:** 10.7150/jca.95269

**Published:** 2024-04-29

**Authors:** Xiaoyang Han, Yan Gao, Mei Jiang, Zhefeng Li, Jiahao Guo, Yue Li, Junjie Yi, Lisha Hou, Jin Cheng, Lei Feng, Yulan Jin, Xiaoting Zhao, Wentao Yue

**Affiliations:** 1 Central Laboratory, Beijing Obstetrics and Gynecology Hospital, Capital Medical University, Beijing Maternal and Child Health Care Hospital, Beijing, China.; 2 Department of Pathology, Beijing Obstetrics and Gynecology Hospital, Capital Medical University, Beijing Maternal and Child Health Care Hospital, Beijing, China.

**Keywords:** ovarian cancer, platinum resistance, TACSTD2, prognosis, scRNA-seq

## Abstract

**Purpose:** Platinum-based chemotherapy is effective but limited by resistance in high-grade serous ovarian cancer (HGSOC). Single-cell RNA sequencing (scRNA-seq) can reveal tumour cell heterogeneity and subclonal differentiation. We aimed to analyze resistance mechanisms and potential targets in HGSOC using scRNA-seq.

**Methods:** We performed 10× genomics scRNA-seq sequencing on tumour tissues from 3 platinum-sensitive and 3 platinum-resistant HGSOC patients. We analyzed cell subcluster communication networks and spatial distribution using cellchat. We performed RNA-seq analysis on TACSTD2, a representative resistance gene in the E0 subcluster, to explore its molecular mechanism.

**Results:** Epithelial cells, characterized by distinct chemotherapy resistance traits and highest gene copy number variations, revealed a specific cisplatin-resistant cluster (E0) associated with poor prognosis. E0 exhibited malignant features related to resistance, fostering growth through communication with fibroblasts and endothelial cells. Spatially, E0 promoted fibroblasts to protect tumour cells and impede immune cells infiltration. Furthermore, TACSTD2 was identified as a representative gene of the E0 subcluster, elucidating its role in platinum resistance through the Rap1/PI3K/AKT pathway.

**Conclusions:** Our study reveals a platinum-resistant epithelial cell subcluster E0 and its association with TACSTD2 in HGSOC, uncovers new insights and evidence for the platinum resistance mechanism, and provides new ideas and targets for the development of therapeutic strategies against TACSTD2+ epithelial cancer cells.

## Introduction

Ovarian cancer, which has a 5-year survival rate of under 30%, is a leading cause of mortality among gynaecological malignancies, particularly in advanced high-grade serous ovarian cancer (HGSOC) 1, 2. The primary treatment involves cytoreductive surgery combined with platinum-based chemotherapy, which initially yields high response rates and clinical remission. However, approximately 70% of patients experience relapse, and tumours develop strong resistance to platinum-containing chemotherapy 3, 4. Therefore, there is an urgent need to explore the molecular contributors to cisplatin resistance and develop new targeted treatments for HGSOC.

Single-cell RNA sequencing (scRNA-seq) offers an opportunity to comprehensively decipher transcriptional landscapes and potential targets, enabling dynamic assessments of cell populations within the tumour microenvironment under various treatment conditions 5, 6. Studies have shown that EMT is also closely related to cell resistance. Ovarian cancer cells undergoing EMT simultaneously induce stem-like cell characteristics, which are manifested as increased self-repair and tumorigenic abilities, and increased resistance to cisplatin and paclitaxel 7. A study demonstrated through scRNA-seq that under cisplatin pressure, phenotypically uniform ovarian cancer cells undergo epigenetically driven cellular reprogramming, acquiring new adaptive platinum-resistant states through cellular stress 8. While scRNA-seq enhances the potential target scope, the single-cell-level landscape descriptions for chemotherapy sensitivity and resistance in HGSOC are scarce, despite comprehensive transcriptomic profiling reported in study 9, the thorough screening of potential targets and mechanisms remains unclear.

TACSTD2 (tumour-associated calcium signal transducer 2), also known as Trop2 (trophoblast antigen 2) or GA7331 (gastrointestinal tumour-associated antigen), is a cell surface glycoprotein that has been implicated in cancer metastasis and as a poor prognosis marker 10-13, including ovarian cancer. Furthermore, antibody-drug conjugates targeting Trop2 (TACSTD2), such as Sacituzumab govitecan, have shown therapeutic effects in triple-negative breast cancer 14, indicating that TACSTD2 is a promising therapeutic target. Although TACSTD2 has been reported to be associated with metastasis and a poor prognosis marker in ovarian cancer 15, its relevance to platinum resistance and the underlying mechanism have not been discovered. Therefore, investigating TACSTD2 can not only enhance understanding of the pathogenesis of ovarian cancer but also reveal new therapeutic strategies, particularly for patients who have developed resistance to conventional chemotherapy.

To investigate the cells driving cisplatin resistance in ovarian cancer tissues, we analysed 10× genomics scRNA-seq data and 10× spatial transcriptome data from HGSOC tissues to identify platinum-resistant cell clusters and explore resistance mechanisms. We discovered a platinum-resistant epithelial cell subcluster E0, which was associated with poor prognosis and malignant features, and communicated with fibroblasts and endothelial cells to foster tumour growth and immune evasion. We also revealed that TACSTD2, a representative gene of the E0 subcluster, was a resistance gene that mediated platinum resistance in ovarian cancer through the Rap1/PI3K/AKT pathway. Our findings provide new insights and evidence for the platinum resistance mechanism of HGSOC, and offer new ideas and targets for the development of therapeutic strategies against TACSTD2+ epithelial cell subclusters, lay a profound foundation for the future use of TACSTD2 antibodies in the treatment of ovarian cancer.

## Methods and Materials

### Collection of ovarian cancer samples

The patient was diagnosed with high-grade serous ovarian cancer with metastasis; stage: IIIC; CA199 (U/ml): 7.04; CEA: 1.98; CA125: 272; CK7, WT1, PAX8, and PR: positive. Tissue samples were procured from ovarian cancer patient, freshly collected during resection and transported on ice with MACS Tissue Storage Solution. Subsequently, tissues underwent 2-3 phosphate-buffered saline washes and ice mincing. Dissociated cells, obtained through 2 min 0.25% trypsin digestion, were loaded following the standard protocol of the Chromium single-cell 3' kit, capturing 5,000 to 10,000 cells/chip position (V3 chemistry). scRNA-seq libraries were generated using the 10× Genomics Chromium platform and sequenced on an Illumina NovaSeq 6000 system. After the patient's initial treatment with cisplatin, a follow-up of the treatment history indicated that the ovarian cancer recurred after 6 months, leading to a diagnosis of cisplatin-sensitive ovarian cancer 16. The study adhered to the Declaration of Beijing Obstetrics and Gynaecology Hospital and received approval from the local medical ethics committee (approval number: 2018-KY-048-01).

### Data acquisition

The scRNA-seq data of sensitive1 and GSE154600 (2 sensitive and 3 resistant tissues) were analysed in combination. The scRNA-seq data of 5 normal and 7 HGSOC tissues were obtained from GSE184880. And the spatial transcriptome data were obtained from GSE211956 (5 tissues). Clinical data of 23 samples was shown in table S1.

Additional bulk RNA-seq and clinical information were acquired from The Cancer Genome Atlas (TCGA) dataset, GTEx dataset (https://gtexportal.org/home/datasets) and GDSC dataset were utilized (Table S2).

### scRNA-seq analysis

scRNA-seq data were processed and analysed using the Seurat (v 4.1.1) R package. The gene-cell matrix was generated and analysed using the Seurat software. Cells expressing fewer than 300 genes were considered low quality and excluded. Additionally, cells with more than 5000 expressed genes or mitochondrial transcripts exceeding 15% of total transcripts were removed. Post quality control and normalisation, the top 2000 highly variable genes were selected for Principal Component Analysis (PCA). Uniform Manifold Approximation and Projection (UMAP) was constructed using principal components. Canonical Correlation Analysis (CCA) via the Seurat package was applied to eliminate batch effects and perform major clustering. Marker genes were calculated using the Wilcox test algorithm (log2FC > 0.5, p <0.05, min.PCT=0.25), and cell types were defined based on biomarker expression.

### RNA-seq

To investigate the cisplatin resistant mechanism of TACSTD2, SKOV3 cells were divided into four groups: sh NC (negative control), sh TACSTD2, shNC+DDP (cisplatin treatment), and sh+DDP (TACSTD2 knockout and cisplatin treatment). The cisplatin treatment condition involved exposing cells to 10 μg/ml of cisplatin for 48 hours. Following the treatment, total RNA was extracted for RNA-seq analysis.

Sample quality assessment was conducted by Annoroad using two methods: NanoPhotometer® UV-Vis spectrophotometry (Thermo Fisher) to assess sample purity, and the Agilent Technologies 2100 RNA Nano 6000 Assay Kit to evaluate RNA sample integrity and concentration. Upon confirming the quality of total RNA samples, purification of mRNA was performed based on the characteristics of different sources. High-quality libraries were prepared and sequenced using the Illumina platform. The sequencing strategy employed was paired-end (PE) 150.

### Functional enrichment analysis and cluster scoring

Gene set functional analysis aimed to explore clustering and shared functions of gene sets in specific biological processes, pathways, and functions. Using the 'clusterProfiler' tool, we associated these gene sets with our single-cell RNA-seq data to identify functional enrichment across different cell types or conditions.

AUCell quantified the degree of gene set enrichment by calculating the area under the curve of the enrichment score for each gene set in every cell. We utilized the 'c2.cp.kegg.v7.5.1.symbols.gmt' and 'c5.all.v2023.1.Hs.symbols.gmt' gene set collections from the Molecular Signatures Database (https://www.gsea-msigdb.org/gsea/msigdb/).

### Pseudotime trajectory analysis

Pseudotime trajectory construction was performed using the R package 'monocle3' (version 1.0.1). We employed the UMAP method for dimensionality reduction and visualized the pseudotime trajectory using the 'plot_cells' function.

### Single-cell Copy Number Variation (CNV) assessment

The interplay between malignant and non-transformed cells constitutes the tumour microenvironment. Using the 'inferCNV' R package, we identified cells with clonal large-scale chromosomal copy number variations as malignant cells. T cells and myeloid cells were used as controls to infer copy numbers. The threshold parameter for 'inferCNV' was set at 0.1, with all other parameters at their default values. Finally, denoising was performed to generate a copy number profile, with epithelial cells considered as malignant cells.

### Survival analysis

For each cell subcluster, the top 10 marker genes were extracted. Subsequently, using GSVA, subcluster-specific feature scores were calculated for 379 ovarian cancer patients from the TCGA cohort. These feature scores were then combined with overall survival data, and Kaplan-Meier survival analysis was performed using the 'survival' R package.

Survival analysis specific to platinum-treated ovarian cancer patients was conducted using the online tool (https://kmplot.com/analysis/). The analysis was performed using the 'Use multiple genes' option.

### Cell communication

Cell communication analysis was performed using the 'CellChat' R package (version 1.1.3). The analysis utilized the CellChat package. The 'hierarchy' function was employed to visualize the signalling outputs across all cell types.

### WGCNA analysis

To identify the key gene modules of TACSTD2 causing cisplatin resistance. Take the data from fpkm for analysis (c.solargenomics.com), including shNC, shNCDDP, shDDP. Set the soft threshold to 13. More details were described in previous studies 17.

### Prediction of cisplatin sensitivity

RNA-seq data from 376 ovarian cancer samples with clinical information were obtained from The Cancer Genome Atlas (TCGA) dataset (https://portal.gdc.cancer.gov). Using the Cancer Rx Gene Database, a prominent publicly available resource for platinum genomics (https://www.cancerrxgene.org/), we predicted chemotherapy responses for each sample through the pRRophetic package in R.

### Cell culture and transfection

Human ovarian cancer cell lines (SKOV3, A2780, CAOV8, CAOV3) as well as ovarian surface epithelial cells (OSE) were cultured using previously established protocols and authenticated by the STR method 18. SKOV3 and CAOV3 were used for specific experiments. For viral transfection, cells were seeded in 96-well plates and exposed to stable transfection virus along with infection-enhancing reagent A. After 24 hours of transfection, the medium was replaced and puromycin was added for selection twice. Subsequently, qRT-PCR and Western blot analyses were performed to assess the efficiency of gene knockout. Target sequences are provided in the supplementary material (Table S3).

For Rap1 plasmid transfection, the lipofectamine 3000 protocol was followed. Detailed procedures can be found in the supplementary materials.

### Western blot and qRT-PCR

Western blot and qRT-PCR were conducted following established procedures described in previous studies. The specific primer sequences for qRT-PCR and antibody information for western blotting are provided in the supplementary material (Table S3).

### Flow cytometry analysis

Flow cytometry analysis was performed following established protocols outlined in previous studies 18. Cells were treated with a final concentration of 10 μg/ml of cisplatin for 48 h. The BD FACSCanto and Beckman Coulter instruments were used.

### Spheroid formation assay

Spheroid formation assays were conducted under serum-free and nonadherent conditions. The cells were seeded at a density of 100 cells/well in 48-well low-adhesion plates with serum-free dual-antibiotic-containing DMEM. The following day, cisplatin was added to the culture medium at a final concentration of 10 μg/ml. After 10 days, spheroids exceeding 50 μm were counted, and statistical analysis evaluated the results.

### CCK-8, wound healing, invasion assay

Following the protocols established in previous studies 18.

### Statistical analysis

All experiments were independently repeated at least three times, and statistical differences between experimental groups were assessed using Student's t-test. GraphPad Prism 8.0.2 software and R 4.1.2 was employed for statistical analysis. Statistical significance was denoted as *p<0.05, **p<0.01, and ***p<0.001.

## Results

### Single-cell landscape of platinum resistant and platinum sensitive tissues in HGSOC

**Figure 1:**
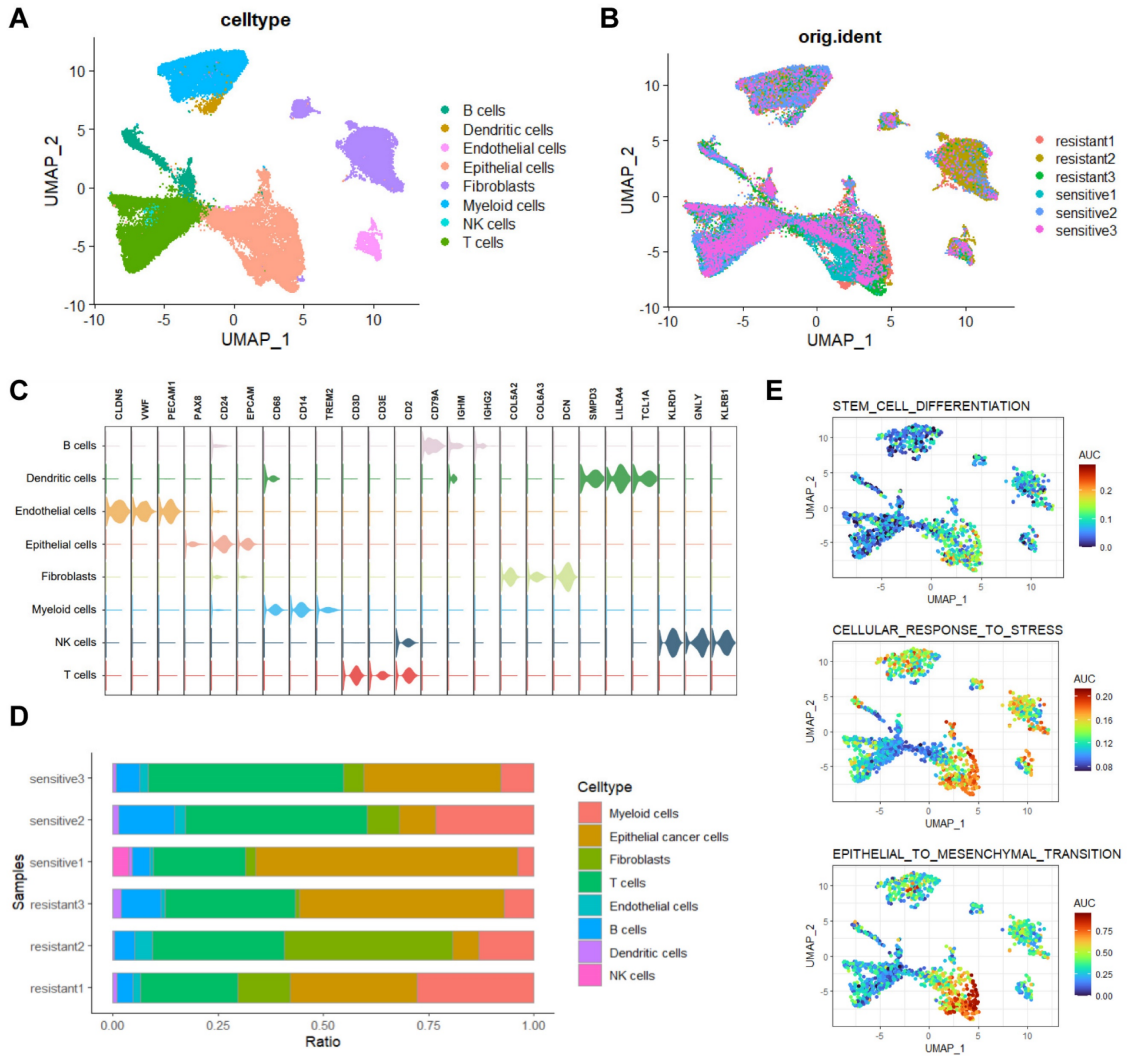
** Single-cell transcriptomic analysis of HGSOC tissues.** A, B. UMAP visualization of cell types in platinum-resistant and platinum-sensitive HGSOC tissues. C. Three representative markers for cell types. D. Proportions of cell types in platinum-resistant and platinum-sensitive HGSOC tissues. E. AUCell analysis of chemo resistant biological enrichments.

We conducted single-cell RNA sequencing (scRNA-seq) on three platinum-resistant and three platinum-sensitive HGSOC tissues, generating a dataset of 51,176 cells after quality control. After batch correction, cell- clustering, and annotation (Figure S1A, B), eight major cell types were identified (Figure 1A, B). Representing annotated cell types from previous research 19: B cells (n=3080, markers: CD79A, IGHM, IGHG2), Dendritic cells (n=501, markers: SMPD3, LILRA4, TCL1A), Endothelial cells (n=1255, markers: CLDN5, VWF, PECAM1), Epithelial cells (n=13549, markers: PAX8, CD24, EPCAM), Fibroblasts (n=8683, markers: COL5A2, COL6A3, DCN), Myeloid cells (n=8277, markers: CD68, CD14, TREM2), NK cells (n=173, markers: KLRD1, GNLY, KLRB1), and T cells (n=15658, markers: CD3D, CD3E, CD2)(Figure 1C). Cell proportion analysis revealed high heterogeneity ratios in platinum-resistant and platinum-sensitive groups (Figure 1D). We next performed AUCell analysis on three characteristics associated with platinum resistance in HGSOC, results indicated significant enrichment of 'stem_cell_differentiation', 'cellular_response_to_stress' and 'epithelial-mesenchymal transition' (EMT) in epithelial cells and lower enrichment in fibroblasts (Figure 1E). The inferCNV results also showed significant differences in epithelial cells compared to other cell types (Figure S1C).

Based on the above findings, epithelial cells had ovarian cancer platinum resistant phenotypes, and the greatest difference in gene copy number variations (CNV) among all cells, prompting the extraction of epithelial cells for in-depth exploration of key resistant subclusters.

### Identification of E0 as a resistant subgroup in epithelial cells

**Figure 2:**
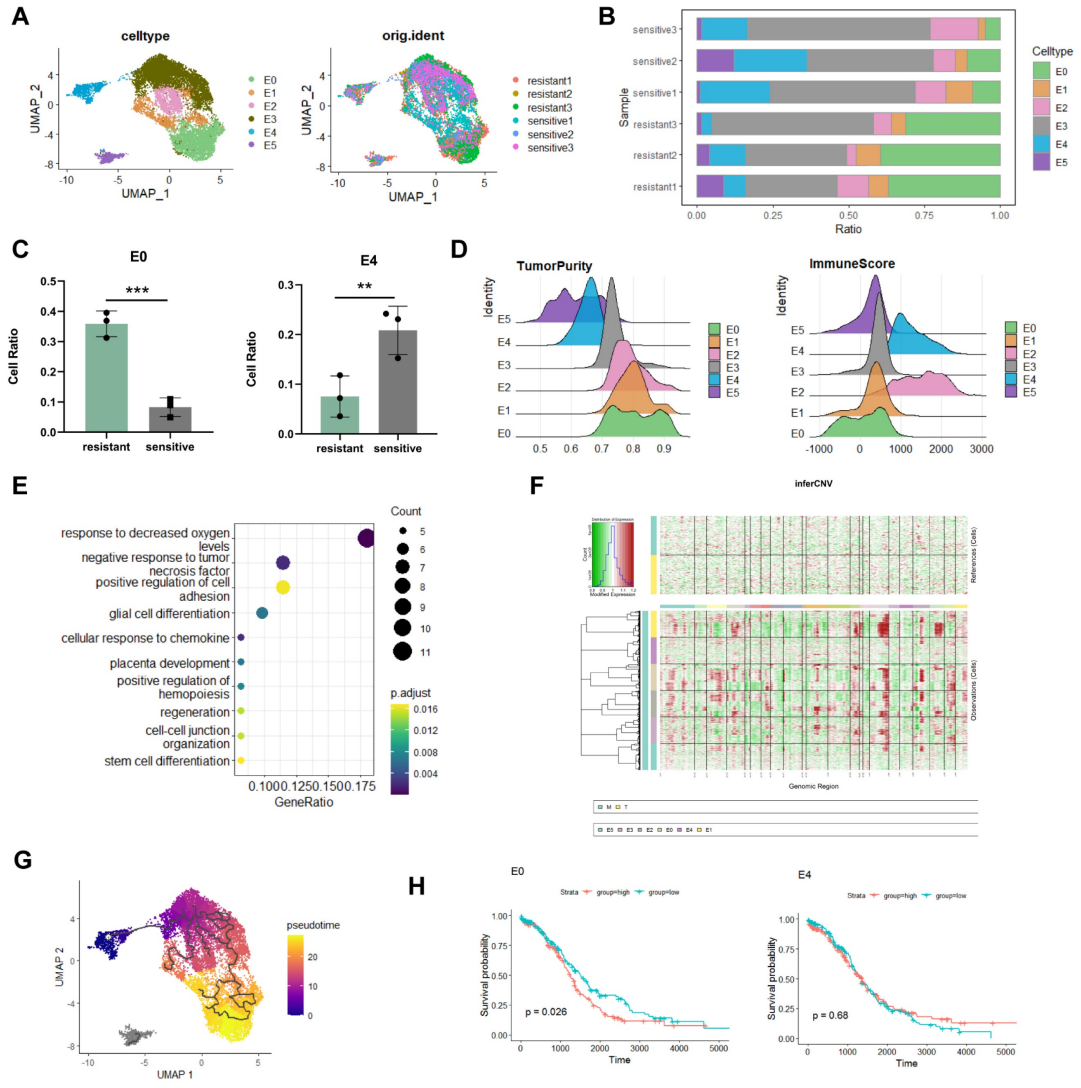
** Identification of distinct epithelial cells subcluster.** A. UMAP visualization of epithelial cells. B, C. Proportion analysis of epithelial cells subcluster. D. Estimate analysis showing the tumour purity and immune score of each epithelial cells subcluster. E. Enrichment analysis of E0. F. InferCNV analysis of epithelial cells. G. Monocle3 trajectory analysis showed E0 at the terminal developmental stage. H. Survival analysis of the E0 and E4 subclusters. (*p<0.05, **p<0.01, ***p<0.001, t-test, log-rank test).

To further determine the key-clusters that lead to platinum resistance, after the extraction of epithelial cells and dimensionality reduction clustering, we divided epithelial cells into six subclusters (Figure 2A). Analysing cellratio highlighted significant differences between platinum-resistant and sensitive groups. The proportion of the E0 subgroup was significantly higher in the platinum-resistant group (36% in resistant; 8% in sensitive), whereas the proportion of the E4 subgroup was considerably lower (7% in resistant; 20% in sensitive) (Figure 2B, C). E0 was higher in resistant tissues suggested that E0 played an important role in chemotherapy resistance. E0 exhibited higher tumour purity and lower immune scores in estimate analysis (Figure 2D), indicative of challenging treatment conditions 20. Enrichment analysis unveiled E0's adaptation to hypoxic levels and a negative response to tumour necrosis factor (Figure 2E), hypoxia increases tolerance to drug toxicity, induces high expression of platinum-resistant genes in cancer cells 21, and suggested that E0 was related to platinum resistance. To better investigate the roles of E0 in epithelial cells, inferCNV showed that E0 displayed the highest gene CNV among all epithelial subgroups, further supported by pseudotime trajectory analysis, positioning E0 at the late stage of tumour cell differentiation with E4 as the starting point (Figure 2F, G). Along trajectory, late stage of epithelial subclusters indicate tumour cells' metastatic, treatment resistant characteristics 17.

To check the relationship of epithelial subclusters with ovarian cancer prognosis, survival analysis revealed that E0 was associated with poor prognosis in ovarian cancer patients (log-rank p=0.026), whereas E4 exhibited no significant relationship with prognosis (log-rank p=0.68) (Figure 2H). This substantiates E0 as a pivotal subgroup in epithelial cancer cells linked to malignant progression and poor prognosis, related to platinum resistance.

The analysis of each subtype was associated with the prognosis of HGSOC patients receiving platinum-based therapy, and we found that E0 was correlated with a poorer prognosis (log-rank p=0.035), contrasting the insignificance of E4 (log-rank p=0.61) (Figure S2A).

Previous studies reported that EMT, cellular stress, regulation of cell growth, and stem cell differentiation enhanced chemoresistance in ovarian cancer 8, 22, 23. In addition to DNA damage repair, ribosomal biogenesis can promote tumour progression and platinum resistance 24. Through AUCell analysis, we found that EMT, cellular stress, cell growth, stem cell differentiation, DNA damage repair, and ribosomal biogenesis were enriched in the E0 subgroup, signifying its key role in chemotherapy resistance (Figure S2B).

### Crosstalk of E0 in tumour microenvironment

**Figure 3:**
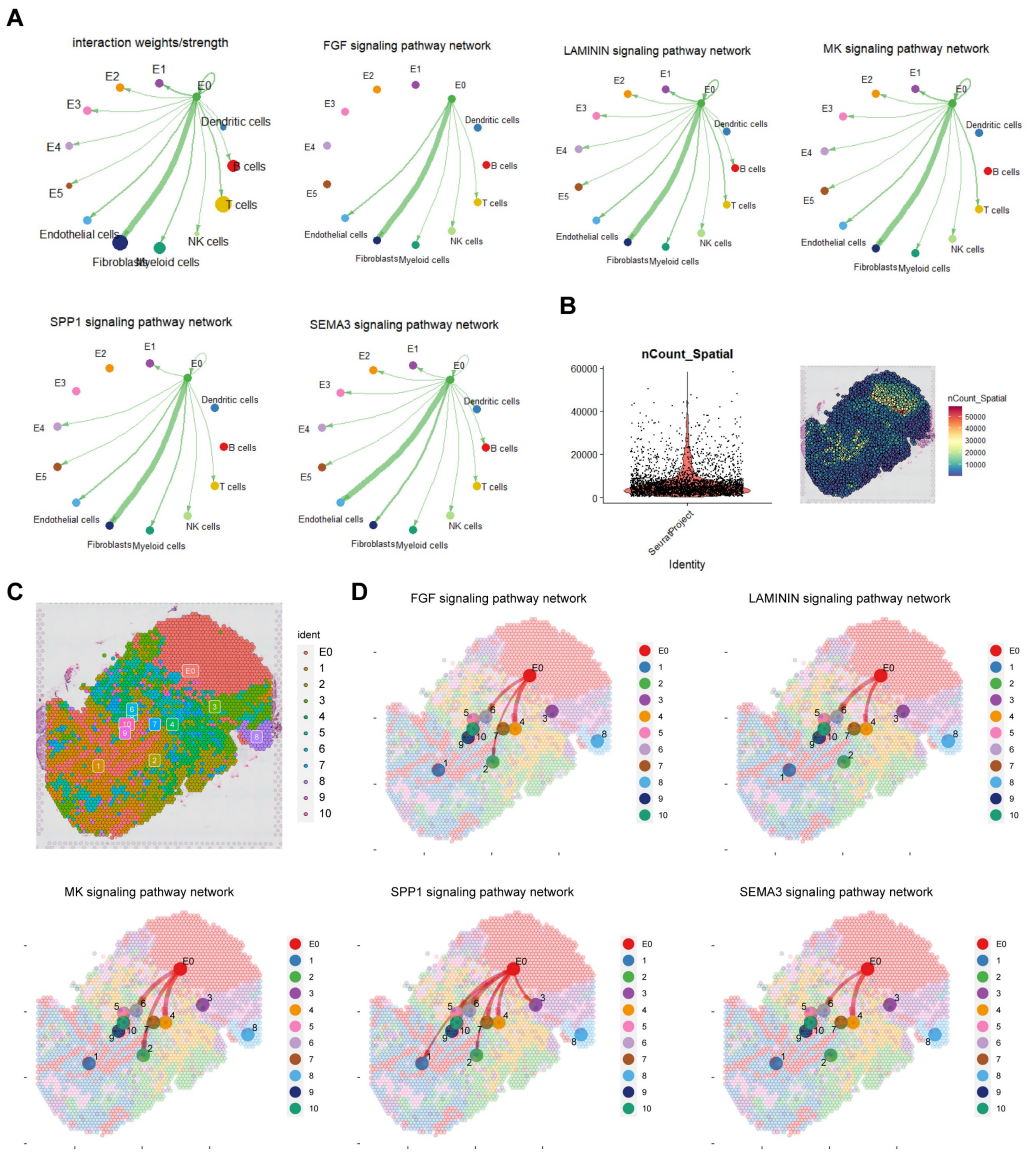
**Cell-cell communication between E0 and TME.** A. Cellchat analysis revealing extensive intercellular communication of E0 and TME. B, C Quality control and annotation of spatial transcriptome. D. Cellchat analysis in spatial transcriptomics.

Cell communication enhances understanding of cellular functions, interactions, and diversity. To explore the connection between the E0 subgroup and tumour microenvironment (TME) we conducted cellchat analysis (Figure 3A; Figure S3A, B). Results revealed fibroblasts and endothelial cells as primary interaction partners with E0, predominately through FGF, LAMININ, MK and SPP1 signalling for fibroblasts and SEMA3 for endothelial cells.

In P8 spatial transcriptome analysis, after quality control, standardization and dimension reduction clustering, divided into 11 clusters including E0, cluster 1 and 3 were mainly immune cells, cluster 2 was mainly fibroblasts, cluster 4 was a mixed group of fibroblasts and cancer cells, cluster 5 was a mixed group of immune cells and erythrocytes, clusters 6 and 10 were mixed groups of fibroblasts and endothelial cells, and cluster 7 was myeloid cells (Figure 3B, C; Figure S3C). Cell communication results from spatial transcriptomics confirmed that E0 interacts with fibroblasts and endothelial cells, mediated by FGF, LAMININ, MK, SPP1 and SEMA3 signalling pathways (Figure 3D).

Considering spatial distribution, E0 was observed to promote fibroblasts and endothelial cells, protecting tumour cells and impeding immune cells (especially myeloid cells) infiltration through FGF, MK, SPP1 and LAMININ pathways, and through SEMA3 pathway to enhance vascular formation.

### The impact of TACSTD2, a representative gene of E0, on ovarian cancer

**Figure 4:**
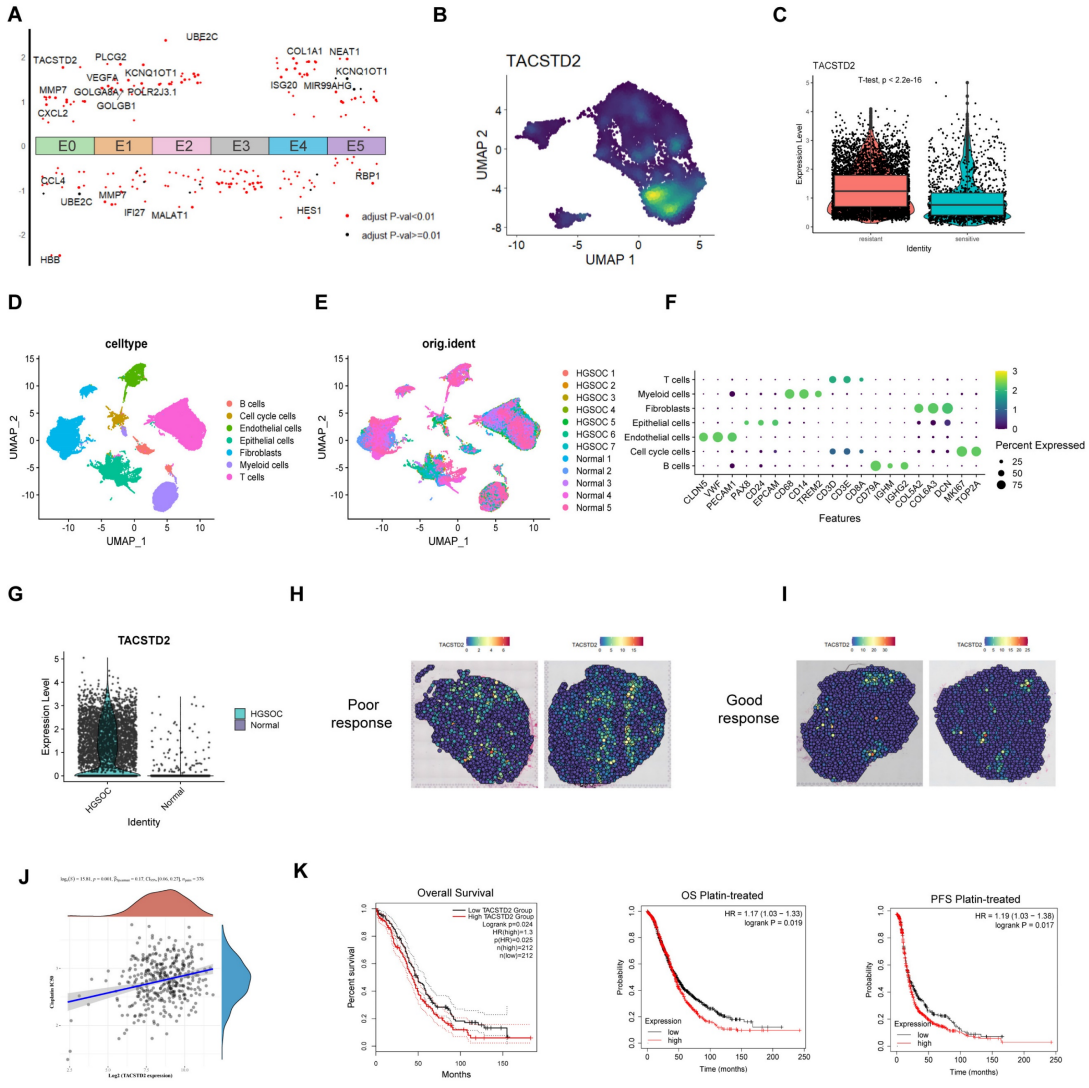
** Overexpression of TACSTD2 in E0 and its impact in ovarian cancer.** A. Top differential genes in each subpopulation in epithelial cells. B. TACSTD2 is a representative gene of E0. C. High expression of TACSTD2 in platinum-resistant ovarian cancers. D-F. Single-cell sequencing analysis of seven HGSOC and five normal tissues. G. Expression of TACSTD2 in epithelial cells in HGSOC and normal tissues. H, I. HGSOC spatial transcriptome analysis of TACSTD2 in good response and poor response to treatment. J. Correlation between TACSTD2 expression and IC50 prediction for cisplatin in ovarian cancer. K. Survival analysis of all patients with ovarian cancer and those receiving platinum-based therapy. (*p<0.05, **p<0.01, ***p<0.001, statistical methods: t-test, log-rank test).

To gain further insights into the pivotal genes driving resistance within the E0 subcluster, by analysing the differential genes of all subclusters, we visualized the highly expressed genes in E0 (Figure 4A; Table S4). Notably, TACSTD2 exhibited the highest differential expression and significant specificity in the E0 subcluster, displaying higher expression in resistant tissues (Figure 4B, C; Figure S4A). These findings suggest that TACSTD2 represents a specific high-expression gene within the E0 subcluster, implying its potential relevance to platinum-based chemotherapy resistance in HGSOC. Subsequently, we investigated TACSTD2 expression in 7 HGSOC cases and 5 normal cases (GSE184880). Following batch removal and dimensionality reduction clustering, we analysed epithelial cells, revealing a high expression of TACSTD2 in HGSOC epithelial cells (Figure 4D-G). Bulk RNA-seq investigating TACSTD2 expression across various cancer indicated significantly elevated expression in ovarian cancer (Tumour=376, Normal=180, p<0.001) (Figure S4B). Spatial transcriptome analysis in HGSOC demonstrated higher expression of TACSTD2 in tissues with a poor response to treatment (Figure 4H, I).

Subsequent analysis explored the correlation between IC50 scores and TACSTD2 expression using Spearman's correlation analysis. The results underscored a positive relationship, revealing that higher TACSTD2 expression in ovarian cancer tissues was associated with elevated IC50 values for cisplatin, suggesting TACSTD2 as a predictor of cisplatin treatment response (Figure 4J). Survival analysis further corroborated these findings, demonstrating that heightened TACSTD2 expression was linked to poor overall survival (OS) and progression-free survival (PFS) in patients treated with platinum-based therapy and in all ovarian cancer patients (Figure 4K).

In this part we identified TACSTD2 as a specifically expressed gene in E0, confirmed to be highly expressed in HGSOC through bulk RNA-seq, scRNA-seq, and spatial transcriptome data. Furthermore, elevated TACSTD2 expression was indicative of an adverse prognosis and cisplatin resistance.

### Enhanced sensitivity of ovarian cancer cells to cisplatin following TACSTD2 knockout

**Figure 5:**
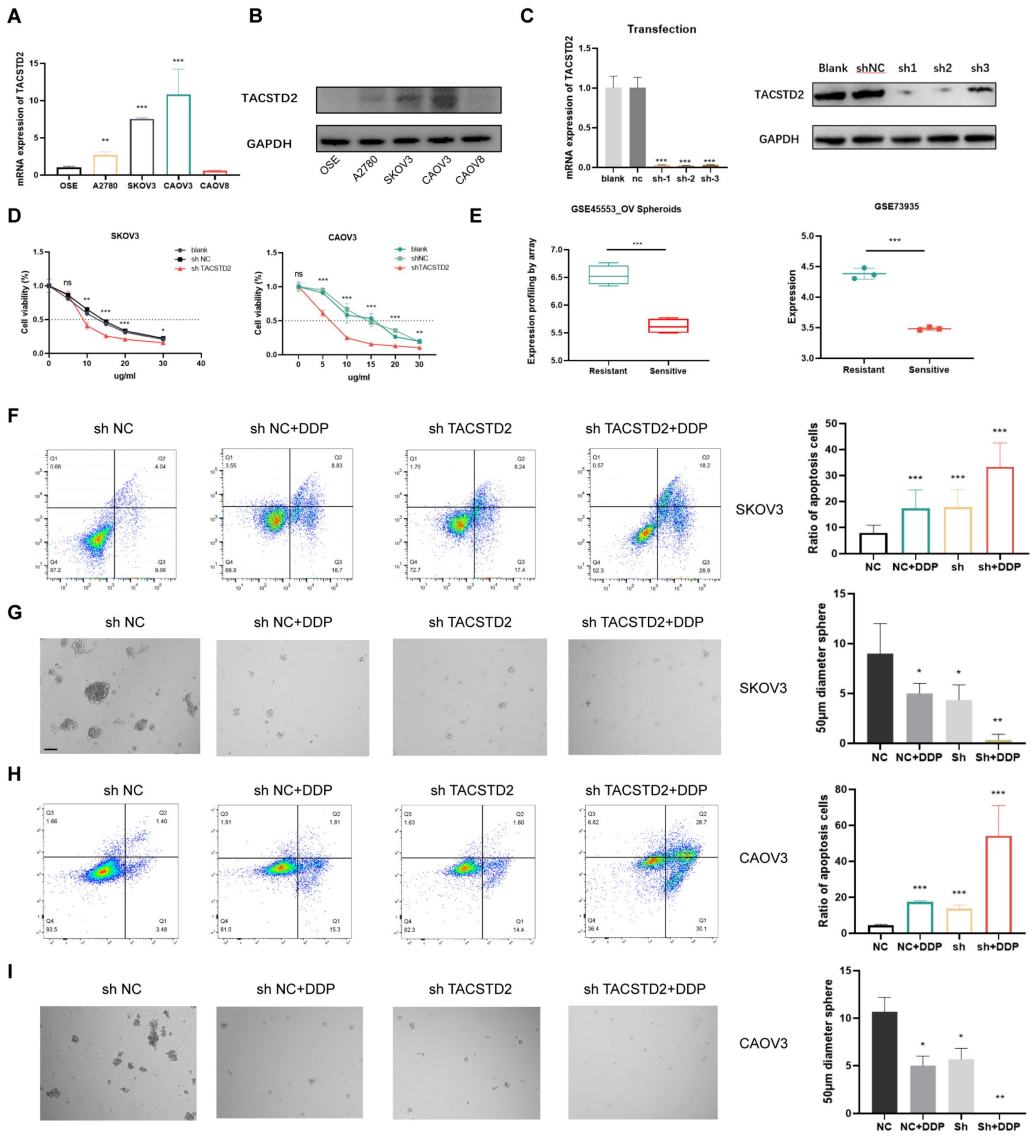
** Knockout of TACSTD2 Enhances Sensitivity to Cisplatin.** A, B. Confirmation of TACSTD2 overexpression in A2780, SKOV3, and CAOV3 cell lines through qRT-PCR and western blotting. C. The Transfection efficiency of TACSTD2 knockout was assessed using qRT-PCR and western blotting. D. Knockout of TACSTD2 reduced cisplatin IC50 values in SKOV3 and CAOV3 cell lines. E. Elevated TACSTD2 expression in platinum-resistant ovarian cancer cell lines compared to that in sensitive cell lines. F, H. Flow cytometry assays indicated increased apoptotic rates in various treatment groups. G, I. Sphere-forming assays assessing the stemness capability of different treatment groups with 100 cells per well. (*p<0.05, **p<0.01, ***p<0.001, statistical methods: t-test).

QRT-PCR and western blotting revealed elevated TACSTD2 expression in A2780, SKOV3, and CAOV3 cell lines (Figure 5A, B). Subsequent functional studies focused on SKOV3 and CAOV3 cell lines. Following TACSTD2 transfection, knockout efficiency was assessed using qRT-PCR and western blotting (Figure 5C). Cisplatin IC50 experiments in SKOV3 and CAOV3 demonstrated that shRNA-mediated TACSTD2 knockout significantly increased sensitivity to cisplatin-induced cytotoxicity. Specifically, the IC50 values decreased significantly, SKOV3 IC50 dropped from 15 μg/ml to 9 μg/ml, while OVCAR3 IC50 decreased from 15 μg/ml to 6 μg/ml (Figure 5D), indicating downregulation of TACSTD2 enhanced the cisplatin sensitivity.

Further analysis of GEO sequencing data for ovarian cancers revealed higher TACSTD2 expression in resistant cells than in sensitive cells (p<0.001) (Figure 5E). Flow cytometry post-cisplatin treatment exhibited increased apoptosis in SKOV3 and CAOV3 compared to the control (NC) group. Interestingly, the TACSTD2 knockout also increased the rate of apoptosis. Moreover, combined treatment with TACSTD2 knockout and cisplatin led to a significant increase in apoptotic rates, signifying enhanced sensitivity of SKOV3 and CAOV3 cells (Figure 5F, H).

The relationship between tumour cell stemness and chemotherapy resistance was confirmed. Tumour stemness, recognized as a principal catalyst for chemotherapy resistance due to its intrinsic resistance to cytotoxic agents, was investigated through sphere-forming assays. SKOV3 and OVCAR3 cells in the control group exhibited strong sphere-forming ability, indicating their strong stem cell characteristics. However, upon cisplatin treatment, there was a notable reduction in the number and size of cell clusters within the spheres. This trend was also observed in the TACSTD2 knockout group. Interestingly, the combined treatment of TACSTD2 knockout and cisplatin resulted in a nearly complete abolition of sphere-forming abilities in SKOV3 and CAOV3 cells, leading to a significant decline in stemness (Figure 5G, I), This demonstrated that TACSTD2 endowed ovarian cancer cells with stem cell-like properties, leading to resistance to cisplatin.

In conclusion, these findings provide further evidence that TACSTD2 knockout enhances the cytotoxic effects of cisplatin.

### TACSTD2-mediated platinum resistance via Rap1/PI3K/AKT Pathway

**Figure 6:**
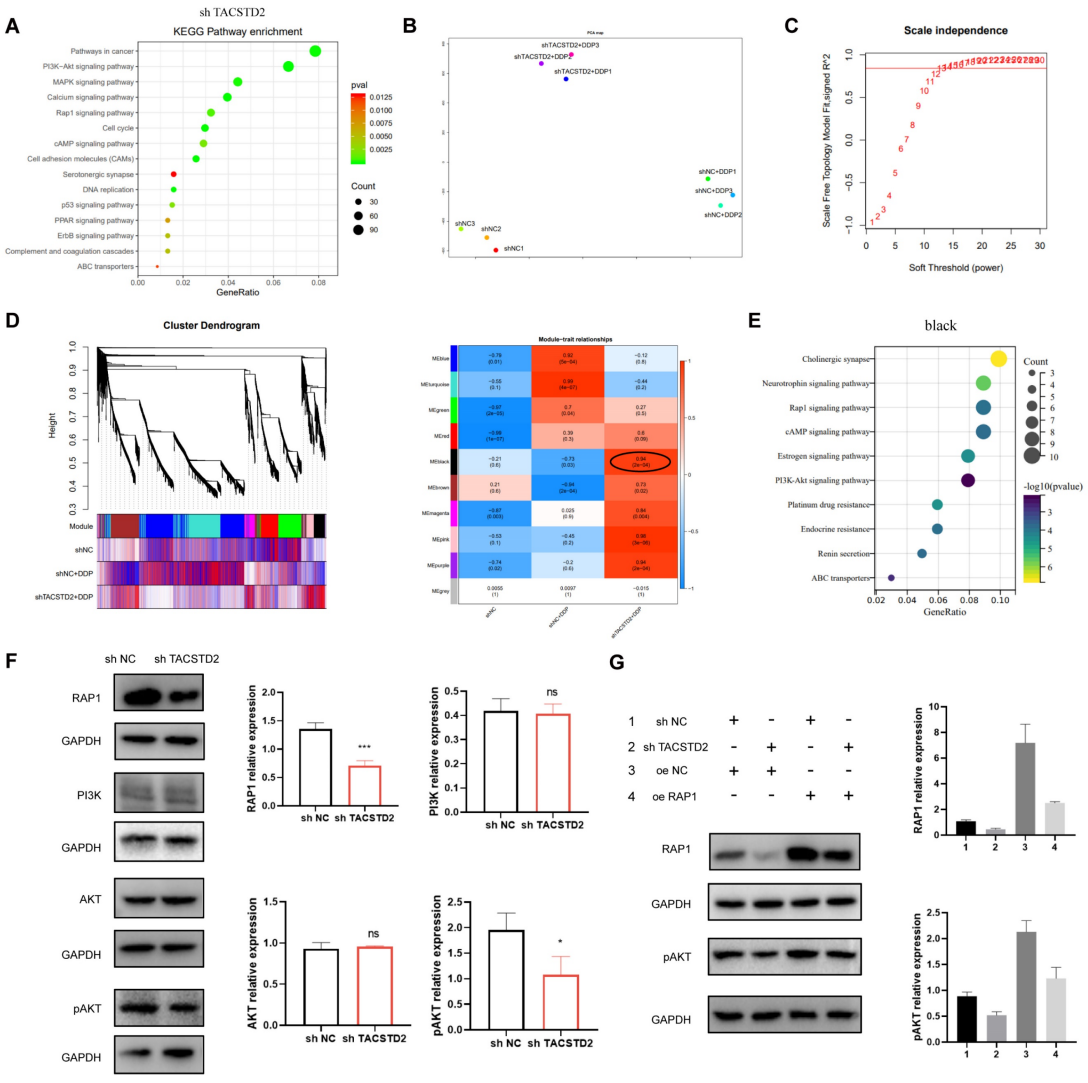
** TACSTD2-mediated Platinum Chemoresistance through the Rap1/PI3K/AKT Axis.** A. KEGG enrichment analysis of differentially expressed genes upon TACSTD2 knockout. B, C WGCNA analysis and soft threshold. D. Major modules associated with TACSTD2 impact on platinum sensitivity in WGCNA analysis (top: correlation coefficient, bottom: p-value). E. Enrichment analysis of the black module. F. Western blot results indicated decreased Rap1 signalling pathway and phosphorylated AKT levels upon TACSTD2 knockout. G. Rescue experiment confirmed TACSTD2 promoted PI3K/AKT signalling via the Rap1 pathway, affecting AKT phosphorylation. (*p<0.05, **p<0.01, ***p<0.001, statistical methods: t-test).

RNA-seq analysis revealed that TACSTD2 primarily enhances the biological functions of ovarian cancer cells through the PI3K-AKT, MAPK, Calcium, Rap1 signalling pathways (Figure 6A). To explore the heightened sensitivity of ovarian cancer cells to platinum-based treatment upon TACSTD2 knockout, we performed PCA dimensionality reduction and WGCNA analysis on sequencing data from the three groups: shNC, shNC+DDP, and shTACSTD2+DDP and soft threshold set to 13 (Figure 6B, C). The results highlighted that the black (R=0.94, p=2e-04), pink (R=0.98, p=3e-06), and purple modules (R=0.94, p=2e-04) were most strongly correlated with the shTACSTD2+DDP group (trait) (Figure 6D). Using WGCNA, we identified three modules related to shTACSTD2 combined with cisplatin treatment. Subsequently, gene enrichment analysis was conducted for three modules revealed the black module's functions and pathways such as the Rap1 signalling pathway and platinum resistance (Figure 6E; Figure S4C). Therefore, the black module was identified as the most correlated with platinum resistance.

In-depth analysis integrated TACSTD2-regulated pathways with the Rap1 signalling pathway, focusing on genes associated with platinum resistance and the Rap1 signalling pathway as per KEGG maps. Based on this analysis, we found that TACSTD2 modulates the PI3K/AKT pathway through the Rap1 signalling pathway, thereby influencing AKT phosphorylation, and subsequently influencing ovarian cancer cell sensitivity to platinum-based treatment (Figure S4D).

Western blotting confirmed that TACSTD2 knockout, compared to the control (NC), resulted in reduced Rap1 expression and decreased levels of phosphorylated AKT, while PI3K and AKT expression remained unchanged. Additionally, Rap1 overexpression increased phosphorylated AKT expression (Figure 6F). Rescue experiments indicated that upon TACSTD2 knockout, Rap1 overexpression restored phosphorylated AKT levels (Figure 6G).

Our combined analysis and experimental results suggested that TACSTD2 regulates the PI3K/AKT pathway through the Rap1 signalling pathway in ovarian cancer cells, thereby modulating AKT phosphorylation and influencing the cytotoxicity of platinum-based treatment in ovarian cancer cells.

## Discussion

Ovarian cancers, particularly HGSOC, exhibit significant heterogeneity, Intratumoral heterogeneity is one of the biggest obstacles to research on high-grade serous ovarian cancer 25, 26. Among chemotherapy-resistant tissues, the content of epithelial cells varied significantly among the three patients, mirroring observations in sensitive tissues. Although some studies have shown that fibroblasts can be used for chemotherapy 27, 28, using AUCell and inferCNV, we identified epithelial cells as the predominant cell type associated with treatment failure, followed by fibroblasts because of the features of epithelial cells and highest copy number variations. Upon reanalysis of epithelial cells, we observed a substantial increase in the proportion of E0 cells in chemotherapy-resistant tissues. Subsequent analyses revealed that E0 exhibited the highest tumour purity and the lowest immune score, suggesting an immune-desert characteristic, rendering it challenging to therapy. Additional features of E0, such as adaptation to hypoxia and the highest gene CNV, underscores its pivotal role in chemotherapy resistance.

Cisplatin-based chemotherapy, a cornerstone in cancer treatment, faces widespread resistance in HGSOC 29. Previous studies reported that stem cell differentiation, cellular stress, and EMT could lead to platinum-based chemoresistance in ovarian cancer. Other research suggested inhibiting ribosome biogenesis, specifically rRNA modification and DNA repair, as a visible approach to sensitize tumour cells to classical cytotoxic chemotherapy 30. AUCell analysis emphasized the enrichment of these biological behaviours in the E0 subgroup, implicating it as a pivotal contributor to chemotherapy resistance in epithelial cells.

Spatially, cell communication showed that E0 interacted primarily through the FGF, LAMININ, MK, SPP1, and SEMA3 signalling pathways. Previous studies reported that SPP1+fibroblasts caused platinum resistance in liver cancer 31, in our study, we found that E0 interacted intensively with fibroblasts through the SPP1 signalling pathway, revealing that E0 induced cisplatin resistance through fibroblasts. In lung cancer, fibroblasts interact with tumour cells through LAMININ signalling, fibroblasts construct an invisible "wall" to protect epithelial cancer cells, inhibit immune cells infiltration and reduce the efficacy of PD-1 antibody drugs 32. These results suggested that E0 promotes fibroblast growth through FGF and modulates fibroblasts through LAMININ, protecting tumour cells and hindering immune cells infiltration in HGSOC.

Further analysis revealed TACSTD2 as a representative gene in the E0 subgroup. The identification of TACSTD2 as a potential contributor to platinum resistance signifies a significant advancement in understanding HGSOC biology. Although TACSTD2 promotes the malignant progression of various cancers, including ovarian cancer, no studies have reported that TACSTD2 causes cisplatin resistance in ovarian cancer. Herein, we demonstrated that elevated TACSTD2 expression leads to cisplatin resistance in ovarian cancer cells through the Rap1/PI3K/AKT signalling pathway.

TACSTD2 (Trop2) spans the cell membrane, featuring extracellular, transmembrane, and intracellular domains, as well as a cytoplasmic tail essential for signal transduction. It primarily promotes tumour cell growth, proliferation, and metastasis by modulating calcium ion signalling pathways, the expression of cell cycle proteins, and reducing fibronectin adhesion 33. TACSTD2 is highly expressed in many cancers, and exhibits differential expression in certain normal tissues. This differential expression and its role in promoting tumour progression make it an attractive new target for the development of antibody-drug conjugates. While TACSTD2 has shown therapeutic progress in breast cancer 34, 35, our single-cell transcriptome analysis revealed its limited feasibility for treating ovarian cancer. TACSTD2 enrichment in E0 cells, not all epithelial cancer cells (Figure S5), suggests it may not have good therapeutic effects as a single target. Therefore, exploring the expression and mechanism of TACSTD2 provides valuable insights for future ovarian cancer treatments.

## Conclusion

In this study, we initially analysed single-cell sequencing data from 6 cases of HGSOC tissue, identifying a predominant subset of platinum-resistant E0 subgroups in the epithelial cells. Among these, E0 emerged as a more proportion in resistant tissues. E0 not only exhibited the highest level of CNV but also displayed malignant biological characteristics, correlating with patient prognosis and platinum resistance. Through single-cell sequencing and spatial transcriptomic analysis, we uncovered interactions primarily between E0 and fibroblasts, and endothelial cells. Notably, fibroblasts seemed to play a protective role by impeding immune cells infiltration and the deterioration of E0. Among represented gene in E0, TACSTD2 has been confirmed to be highly expressed in various cancers. Our research elucidated that elevated expression of TACSTD2 leads to resistance to cisplatin and delineates its resistance mechanism mediated through the Rap1/PI3K/AKT signalling pathway in HGSOC. The findings of this study provide a theoretical basis for the combination of TACSTD2 antibodies with cisplatin, in the treatment of ovarian cancer.

## Supplementary Material

Supplementary figures and tables 1-3.

Supplementary table 4.
