# Supplementary material for: Single-cell and spatial transcriptome sequencing uncover a platinum-resistant cluster overexpressed TACSTD2 in high-grade serous ovarian cancer
Source: J Cancer. 2024 Apr 29;15(11):3427–40. doi: 10.7150/jca.95269 (PMC11134433; doi:10.7150/jca.95269)
Supplement: Supplementary file 1 — Supplementary figures and tables 1-3. [file jcav15p3427s1.pdf]

Supplymentary Figure Legend

Figure S1. Single cell sequencing landscape

A. Quality control of single cell sequencing samples.

B. Dimension Reduction Clustering.

C. InferCNV analysis of all cell types.

Figure S2. Platinum resistant features in epithelial cells

A. Survival analysis of E0 and E4 subclusters in ovarian cancer patients receiving platinum-based therapy.

B. AUCell analysis of the epithelial cells subcluster.

Figure S3. Cell communications with E0 and TME

A. The number of communicate counts of cell type.
B. E0 receptor ligand for fibroblast endothelial cell communication.
C. E0 in spatial transcriptome analysis was identified with TACSTD2.

Figure S4. Effect of TACSTD2 on ovarian cancer

A. UMAP visualization of top genes in E0.
B. Expression of TACSTD2 in various cancers.
C. KEGG of Pink and Purple modules.
D. TACSTD2 promotes PI3K/Akt in ovarian cancer.

Figure S5. Expression of TACSTD2 and epithelial cancer cells markers in HGSOC tissues.

Supplementary FigureS1

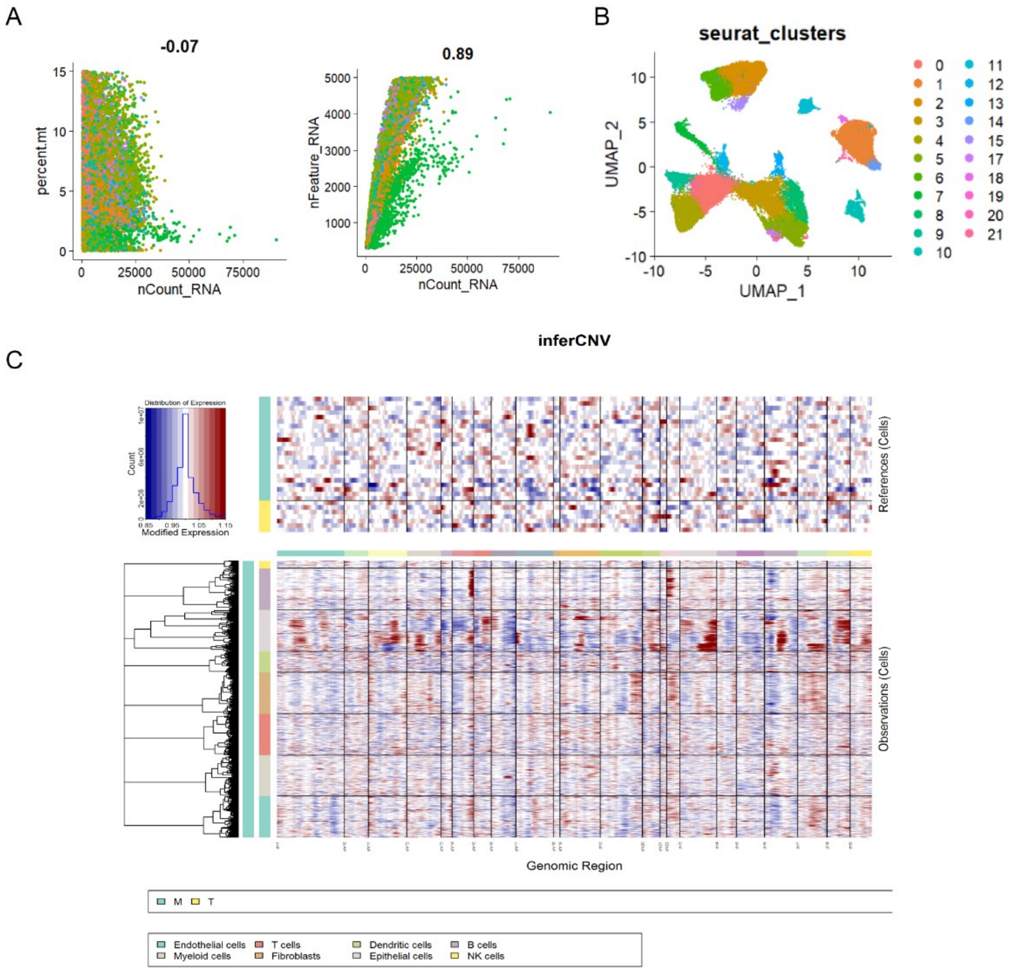

Supplementary FigureS2

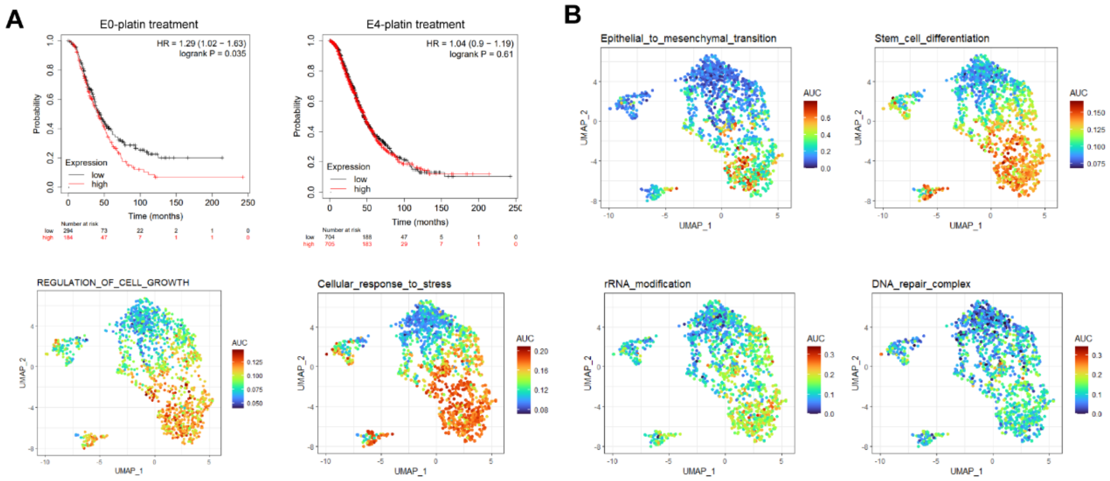

Supplementary FigureS3

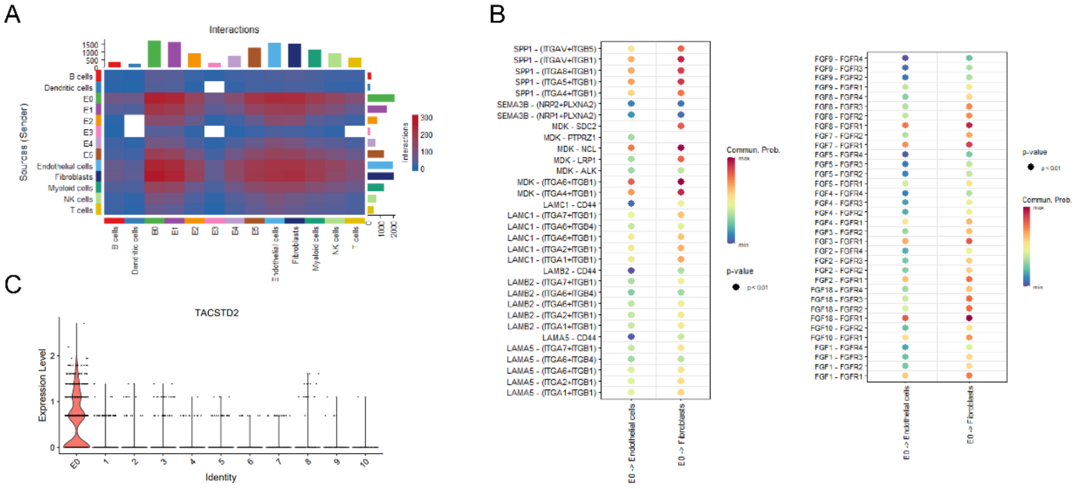

Supplementary FigureS4

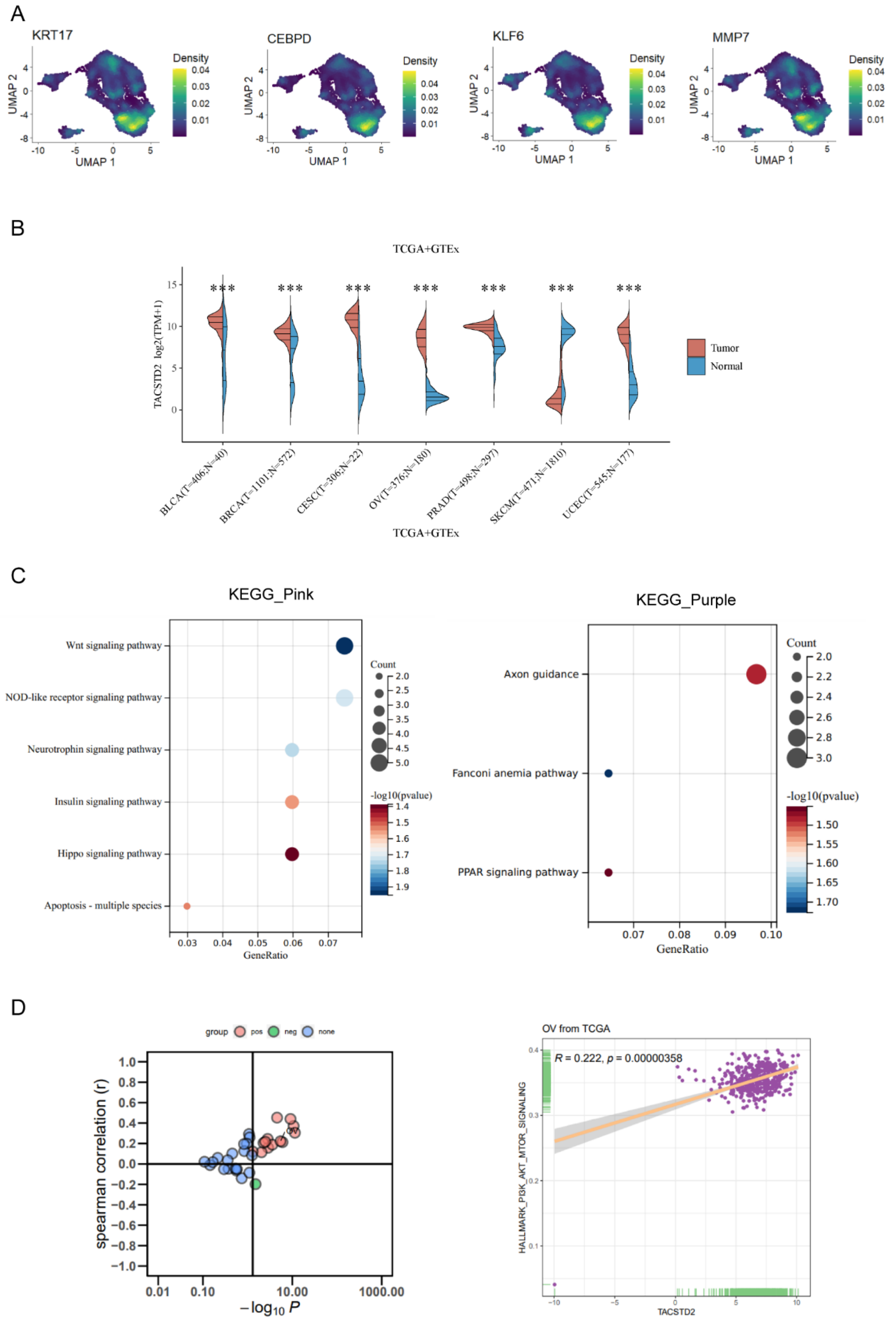

Table S1

Clinical characteristics and summary information of scRNA-seq samples

Samples	Pathologic diagnoses	FIGO stage	Clinical	Access number
Sensitive1	HGSOC	IIIc	Chemotherapy sensitive	PRJNA756768
Sensitive2	HGSOC	IV	Chemotherapy sensitive	GSE154600
Sensitive3	HGSOC	IVa	Chemotherapy sensitive	GSE154600
Resistant1	HGSOC	IV	Chemotherapy resistant	GSE154600
Resistant2	HGSOC	IIIc	Chemotherapy resistant	GSE154600
Resistant3	HGSOC	IVb	Chemotherapy resistant	GSE154600
Normal1	Mucinous cystadenoma of ovary			GSE184880
Normal2	Uterine leiomyomas, ovarian cysts			GSE184880
Normal3	Ovarian endometrotic cyst			GSE184880
Normal4	Adenomyosis			GSE184880
Normal5	Uterine leiomyomas, ovarian cysts			GSE184880
HGSOC1	HGSOC	IIIb	BRCA2 mutation	GSE184880

HGSOC2	HGSOC	IIb	ATM/BRIP1mutation	GSE184880
HGSOC3	HGSOC	Ic	wild-type	GSE184880
HGSOC4	HGSOC	Ic	wild-type	GSE184880
HGSOC5	HGSOC	IIb	BRCA1 mutation	GSE184880
HGSOC6	HGSOC	IIIc	wild-type	GSE184880
HGSOC7	HGSOC	Ic	wild-type	GSE184880
Poor response1	HGSOC	N/A	Chemotherapy resistant	GSE211956 (GSM6506110)
Poor response2	HGSOC	N/A	Chemotherapy resistant	GSE211956 (GSM6506116)
Good response1	HGSOC	N/A	Chemotherapy sensitive	GSE211956 (GSM6506114)
Good response2	HGSOC	N/A	Chemotherapy sensitive	GSE211956 (GSM6506112)
P8	HGSOC	N/A	Chemotherapy resistant	GSE211956 (GSM6506117)

HGSOC: High grade serous ovarian cancer.

Sample P8, used for cell communication analysis.

Analysis of cellratio in resistant and sensitive tissues, TACSTD2 expression between normal and HGSOC: *t*-test.

Table S2. List of public datasets used in the study

The Cancer Genome Atlas (TCGA) OC datasets

Data	Cancer	Download
RNA-seq	Ovarian Cancer (OV)	UCSC Xena
RNA-seq	Bladder Cancer (BLCA)	UCSC Xena
RNA-seq	Breast Cancer (BRCA)	UCSC Xena
RNA-seq	Cervical Cancer (CESC)	UCSC Xena
RNA-seq	Prostate Cancer (PRAD)	UCSC Xena
RNA-seq	Melanoma (SKCM)	UCSC Xena
RNA-seq	Endometrioid Cancer (UCEC)	UCSC Xena
RNA-seq	Ovary	GTEX

IC50 score, survival and TACSTD2 expression

Analysis	Samples	database	Download
IC50	376	UCSC Xena	https://xenabrowser.net/datapages
IC50	376	GDSC	https://www.cancerrxgene.org
Survival	424	GDC OV	https://xenabrowser.net/datapages
Survival_OS (platinum)	1409	Kaplan-Meier plotter	http://www.kmplot.com
Survival_PFS (platinum)	1259	Kaplan-Meier plotter	http://www.kmplot.com

Table S3
Materials

qRT_PCR

Gene	Primer	
TACSTD2	Forward	CGGCAGAACACGTCTCAGAAG
	Reverse	CCTTGATGTCCCTCTCGAAGTAG
GAPDH	Forward	TGACTTCAACAGCGACACCCA
	Reverse	CACCCTGTTGCTGTAGCCAAA

Western-blotting

Antibody	Make	Dilution
GAPDH	KangChen(KC-5G5)	1:5000
RAP1（RAP1A+RAP1B）	ABclonal(A9725)	1:2000
PI3K	ABclonal(A0982)	1:3000
AKT	CST(#C67E7)	1:3000
pAKT	CST(#9018)	1:2000
Marker	NCM(#9006)	5ul

Transfection

TACSTD2	
sh1	GCACCAGCTCATCGCAGCGT
sh2	CGCACCAGCACACCGACGTC
sh3	CACGCGCTCGTGGACAACGA
